# Integration of functional magnetic resonance imaging and artificial intelligence in Alzheimer’s disease

**DOI:** 10.4103/NRR.NRR-D-25-01816

**Published:** 2026-01-02

**Authors:** Liqin Yang, Yuxin Li, Kuangyu Shi, Axel Rominger, Ruiqing Ni

**Affiliations:** Department of Nuclear Medicine, Inselspital, Bern University Hospital, University of Bern, Bern, Switzerland; Department of Radiology, Huashan Hospital, Fudan University, Shanghai, China; Institute for Regenerative Medicine, University of Zurich, Zurich, Switzerland; Institute for Biomedical Engineering, ETH Zurich & University of Zurich, Zurich, Switzerland

Alzheimer’s disease (AD), the most common cause of dementia, is a progressive neurodegenerative disease characterized by progressive cognitive decline and memory loss. Mild cognitive impairment (MCI) is the prodromal stage of AD, with a conversion rate of 10%–15% per year and 50% conversion rate longitudinally. The pathological features of AD include the aberrant accumulation of amyloid-β plaques, neurofibrillary tangles formed by hyperphosphorylated tau and synaptic dysfunction (Nussbaumer et al., 2025), all of which have cascading effects on the brain activity of AD patients. Functional magnetic resonance imaging (fMRI), specifically blood oxygenation level–dependent fMRI, is a non-invasive and radiation-free technique for indirectly measuring brain activity. It should be noted that fMRI has a double-edged sword nature. While fMRI is a powerful tool for detecting whole-brain-wide alterations in neural activity, its high sensitivity to brain states and complex analysis methods and indices also result in poor reproducibility across studies and compromise its further application in clinical practice. The results derived from extremely limited unpaired samples (e.g., before and after treatment in the same patients) or a longitudinal study design should be further explored.

**Figure 1:**
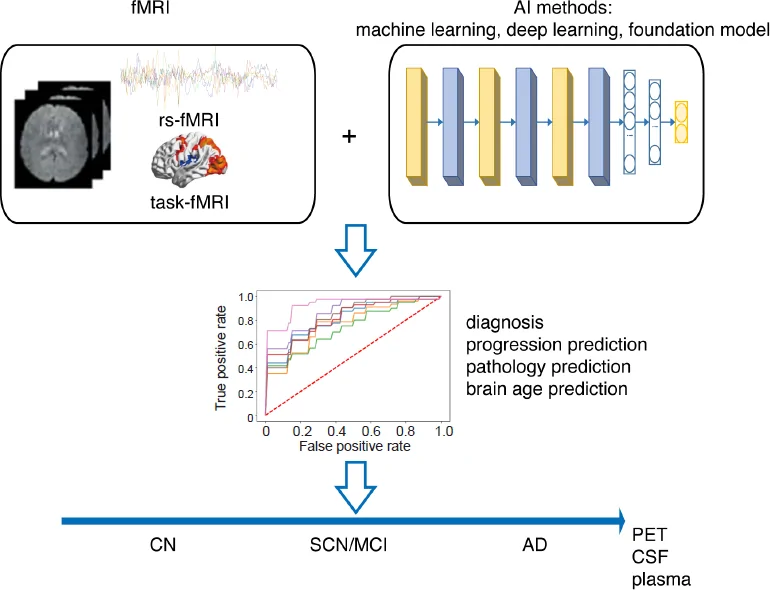
Integration of functional magnetic resonance imaging and artificial intelligence in Alzheimer’s disease. The combination of rs-fMRI and task-fMRI with various AI techniques could be applied to AD to address different classification tasks. Created with Matlab R2024b, BrainNet Viewer 1.7 and Adobe Illustrator 2025. AD: Alzheimer’s disease; AI: artificial intelligence; CN: cognitively normal control; CSF: cerebrospinal fluid; fMRI: functional magnetic resonance imaging; MCI: mild cognitive impairment; rs: resting state; PET: positron emission tomography; SCN: subjective cognitive decline.

In recent years, the integration of fMRI and artificial intelligence (AI) techniques (fMRI-AI) has shown potential in AD diagnosis and progression prediction. AI could enable simplified fMRI analysis and integrate and discover complex features in fMRI signals. Validation on larger, diverse datasets is needed to address issues of overfitting and model generalizability in fMRI-AI studies. Unfortunately, owing to the limited availability of large AD-fMRI datasets in comparison with positron emission tomography and structural MRI datasets, such studies are still rare at present. This perspective highlights recent fMRI-AI findings in AD patients and provides an in-depth analysis of their potential impact on clinical practice and future studies (**Figure 1**).

**fMRI-AI for the diagnosis of AD and its prodromal stages:** AD diagnosis, i.e., the classification between AD patients and cognitively normal controls (CN), is the most frequently probed task in fMRI-AI studies of AD. The availability of public dataset such as Alzheimer’s Disease Neuroimaging Initiative (ADNI) and larger cognitive and neurophysiological differences facilitate AD-CN classification tasks. Furthermore, research has increasingly focused on detecting disease during prodromal stages of AD, such as MCI and subjective cognitive decline. Early detection of this disease before the onset of irreversible clinical symptoms could benefit from timely treatment and intervention, improving the quality of life of patients.

In a systematic review, Warren and Moustafa (2023) investigated 22 articles published before May 2021 that used resting-state fMRI data and deep learning methods to classify AD and its prodromal stages. The included studies used AI methods, including convolutional neural networks, graphical neural networks, autoencoders, and recurrent neural networks. Although most studies were highly dependent on the ADNI cohort and had a limited sample size, these studies had high accuracies ranging between 70% and 98%, demonstrating the effectiveness of the fMRI-AI technique for AD diagnosis.

Foundation models are emerging as new stars in the AI field, in addition to these established deep learning methods. Large language models such as the GPT have demonstrated the great potential of the foundation model framework. Compared with traditional task-based models, foundation models are more general and adaptable. In 2024, the first foundation model for fMRI, Brain Language Model (BrainLM) (Caro et al., 2024), was developed. The BrainLM leverages a Transformer-based architecture to capture the spatiotemporal dynamics in fMRI data. The model was trained on 6700 hours of fMRI data and demonstrated effectiveness in predicting brain states. Most recently, Gao et al. (2025) applied BrainLM for the classification of 95 AD patients and 411 CN patients. They achieved area under the curves (AUCs) of 0.685 and 0.762 under different experimental conditions. Although these results need to be further improved and validated, the foundation models have already gained attention in the fMRI and AD fields.

There are also studies comparing the performance of fMRI with that of other imaging techniques, especially positron emission tomography (PET) imaging. Jann et al. (2025) found that the fMRI-based entropy model matched and even performed better than tau PET model for classifying cognitively impaired (MCI/AD) individuals from normal cognition. Their study was based on 147 subjects (CN/MCI/AD = 95/45/7) from ADNI and 36 subjects (CN/MCI/AD = 19/12/5) from the Estudio de la Enfermedad de Alzheimer en Jalisciences study (EEAJ). Three-dimensional convolutional neural networks were used for model construction. Their model achieved an AUC of 0.73 for the ADNI and an AUC of 0.94 for the EEAJ. These findings suggest that fMRI entropy could be an alternative imaging marker to tau PET for detecting AD-related cognitive impairment.

The fMRI technique has also been applied to predict AD pathology rather than the clinical diagnosis of AD itself. Prakash et al. (2025) built a machine learning model to predict cerebrospinal fluid phospho-tau/amyloid-β 42 (the PATH-fc model), using fMRI data from 289 subjects (CN/MCI/AD=149/109/31) from ADNI. This study provides direct support for the link between fMRI-AI results in AD and its pathophysiology. The authors also noted the low magnitude of the in-sample fit in their results and suggested the need for PET or plasma-based biomarkers of AD pathology in future studies.

The brain clock, which quantifies discrepancies between brain age and chronological age, is also an important aspect of AD-related research. In a recent multi-center study (Moguilner et al., 2024), fMRI datasets from 2953 participants across 15 countries, i.e., seven Latin American and Caribbean countries and eight non-Latin American and Caribbean countries, were analyzed using a deep learning neural network. This fMRI-AI study revealed several brain clock related diversities, including older brain ages in Latin American and Caribbean countries model, an ascending brain-age gap from healthy CNs to MCI to AD, and a larger brain-age gap in females. Brain clock measurements provide an evaluation of brain health for the general population and are thus useful for generating normative brain data and quantitative disease estimation.

**fMRI-AI for MCI-AD progression prediction:** The prediction of MCI-AD progression is important for monitoring disease neurodegeneration but challenging due to the similar status between converters and non-converters at MCI stage. Studies predicting the conversion of MCI to AD based on fMRI-AI are extremely limited due to the lack of longitudinal fMRI data.

Chen et al. (2024) proposed their fMRI-based framework for MCI-AD conversion prediction in 218 MCI patients with 3-year follow-up outcomes from two independent cohorts: Shanghai Memory Study for feature selection and internal cross-validation and the ADNI cohort for external validation. They measured both static and dynamic features of regional activity and functional connectivity and identified several predictors involving the medial temporal lobe, posterior parietal cortex and occipital cortex. For inter-cohort validation purposes, several fMRI analysis methods, including region of interest-based rather than voxel-based indices and group-information guided independent component analysis methods, have been used. They used a traditional AI technique, support vector machine, for model construction and achieved an fMRI-based AUC > 0.76 in both cohorts and AUC > 0.86 when combining fMRI with structural T1-weighted magnetic resonance imaging.

**The potential of task-fMRI-AI application in AD:** While the above-mentioned and most previous fMRI-AI studies focused on resting-state fMRI data, the potential application of task-fMRI in AD has been neglected. Task fMRI could capture brain activation patterns through the localization of functional areas during specific behaviors and intervention stimulation. Task-fMRI data also have a higher signal-to-noise ratio than resting-state fMRI data. In addition, task-fMRI could be more directly involved in the brain‒computer‒interface framework and may be more suitable for clinical use in the future. In recent years, AI-based brain activity decoding and stimuli reconstruction from task-fMRI data have attracted considerable attention. For example, by analyzing cortical fMRI recordings, Tang et al. (2023) reconstructed continuous language that recovers the meaning of perceived speech, imagined speech, and even silent videos. These task-fMRI-AI based non-invasive brain‒computer‒interface techniques may be applied to AD field, for reconstruction of cognition- or memory-related brain activities, which has not been achieved at present.

A notable obstacle in task-fMRI-AI research is the inconsistent task design across different datasets and the difficulty of data acquisition in severe patients. Thus, another hot topic in task-fMRI-AI studies is the prediction of task-fMRI activity from non-task MRI data. Li et al. (2024) proposed a two-stage network model, TS-AI, to synthesize a battery of task contrast maps by leveraging anatomical connectivity and resting-state networks. They also applied their results to AD and identified accelerated shrinkage in the medial temporal and cingulate during disease progression. The synthesis of specific imaging data using another modality could not only be useful for data augmentation but also provide insights into the underlying mechanism of brain dynamics.

**Essential aspects of future fMRI-AI-based multi-center AD research:** Development and validation of fMRI-AI models based on multi-center AD datasets is critical for future research and clinical practice. We suggest that several essential aspects should be considered during this process.

First, the multi-center dataset should satisfy fairness and representativeness to avoid brain clock or AD disease progression differences due to sex, ancestry, etc. And in related studies, bias assessment and subgroup analysis should be performed and reported.

Second, the impact of fMRI preprocessing steps on downstream AI performance could be further studied. At present, most fMRI studies use several similar preprocessing steps, such as skull stripping and motion correction. However, there are also controversial steps, such as global signal regression, which results in reorganization of brain connectivity. In addition, multicenter fMRI harmonization methods, which aim to reduce dataset-specific non-biological variability, should be performed appropriately. Investigation of these steps may help facilitate the establishment of fMRI-AI preprocessing standards and the development of new AI models independent of preprocessing.

Third, indices with different dimensions can be extracted from fMRI data and used as inputs to AI models, but this has not been extensively explored so far. fMRI indices include but are not limited to 1-dimentional time series, 2-dimentional graph constructed by paired functional connectivity, 3-dimentional brain activity or connectivity networks and 4-dimentional original/preprocessed data. The choice, processing, combination and interpretation of these indices would require collaboration of fMRI and AI researchers.

Finally, in the case of restricted data sharing and privacy regulations, AI techniques such as federated learning could serve as a viable alternative. By sharing model parameters rather than the patient’s imaging data, this technique can be used to develop joint model across heterogenous datasets while preserving data privacy and security of each site.

**Conclusion:** The combination of fMRI and AI is a new field in AD research. fMRI-AI could offer valuable information regarding the brain dysfunction that occurs earlier than clinical signs and has shown potential in AD diagnosis and progression prediction. Analysis of both resting-state fMRI and task-fMRI data could further our understanding of AD and provide candidates for future use. At present, the translation of the fMRI-AI results in the clinic is undermined by the small sample size, lack of diverse fMRI datasets and methodological differences in both fMRI and AI analyses. Future studies should involve larger multi-center fMRI datasets, develop more generalized AI frameworks and models, explore their potential in brain‒computer‒interfaces, and pay particular attention to their application in clinical practice.

*This work was supported by China Scholarship Council, No. 202306100073 (to LY) and UniBern Forschungsstiftung, No. 31/2025 (to RN).*
